# Loosenin, a novel protein with cellulose-disrupting activity from *Bjerkandera adusta*

**DOI:** 10.1186/1475-2859-10-8

**Published:** 2011-02-11

**Authors:** Rosa E Quiroz-Castañeda, Claudia Martínez-Anaya, Laura I Cuervo-Soto, Lorenzo Segovia, Jorge L Folch-Mallol

**Affiliations:** 1 Laboratorio de Biología Molecular de Hongos, Centro de Investigación en Biotecnología, Universidad Autónoma del Estado de Morelos. Avenida Universidad 1001 Col. Chamilpa, Cuernavaca 62209, Morelos, México; 2 Instituto de Biotecnología. Universidad Nacional Autónoma de México. Avenida Universidad 2001 Col. Chamilpa, Cuernavaca 62209, Morelos, México

## Abstract

**Background:**

Expansins and expansin-like proteins loosen cellulose microfibrils, possibly through the rupture of intramolecular hydrogen bonds. Together with the use of lignocellulolytic enzymes, these proteins are potential molecular tools to treat plant biomass to improve saccharification yields.

**Results:**

Here we describe a new type of expansin-related fungal protein that we have called loosenin. Its corresponding gene, *loos1*, from the basidiomycete *Bjerkandera adusta*, was cloned and heterologously expressed in *Saccharomyces cerevisiae*. LOOS1 is distantly related to plant expansins through the shared presence of a DPBB domain, however domain II found in plant expansins is absent. LOOS1 binds tightly to cellulose and chitin, and we demonstrate that cotton fibers become susceptible to the action of a commercial cellulase following treatment with LOOS1. Natural fibers of *Agave tequilana *also become susceptible to hydrolysis by cellulases after loosenin treatment.

**Conclusions:**

LOOS1 is a new type of protein with disrupting activity on cellulose. LOOS1 binds polysaccharides, and given its enhancing properties on the action of hydrolytic enzymes, LOOS1 represents a potential additive in the production of fermentable sugars from lignocellulose.

## Background

The central technological impediment to the full industrial utilization of lignocellulose is the general absence of low-cost technology for overcoming its recalcitrance [1]. Filamentous fungi, especially white-rot type basidiomycetes, efficiently degrade plant cell wall biopolymers due to the production of a battery of extracellular enzymes such as cellulases, hemicellulases and ligninases [2]. Basidiomycete fungi represent a source of enzymes with potential applications due to their elevated ligninolytic activity. *Bjerkandera adusta *is a basidiomycete fungus well known for its high ligninase activity [3] and recently, its cellulolytic capabilities have been characterized [4,5].

Plant cell walls are physiologically remodeled by a group of proteins with the ability to relax their components and promote cell enlargement [6]. These proteins, called expansins, are also involved in organogenesis [7], abscission [8], initiation of leaves [9], fruit ripening [10,11], pollen tube penetration of the stigma, and other developmental processes in which cell wall modification occurs [12-14]. It has been proposed that expansins disrupt non-covalent bonds between cellulose microfibrils and matrix polymers by a non-enzymatic mechanism leading to wall loosening and extension [15]. The classification of expansins is based on their phylogenetic relationships. In plants, four families form the expansin superfamily comprising: α-expansin (EXPA), β-expansin (EXPB), expansin-like A (EXLA) and expansin-like B (EXLB). In contrast to EXPA and EXPB, for which experimental data showed cell wall loosening [6,16,17], the function of the EXLAs and EXLBs has been deduced solely from their gene sequences, and to date, only one example of biological activity has been established for a member of the EXLB family, involved in root colonization by a mycorrhizal fungus in tomato plants [18]. Expansin proteins contain between 250-275 amino acids, divided among two domains and a signal peptide. The N-terminal domain (or domain I) acquires a DPBB (*double psi beta barrel*) fold, with structural and sequence similarity to glycoside hydrolase family 45 (GH45) proteins, a β-1,4-endoglucanase family [19,20]. Domain II, at the C-terminus, presents homology to proteins of the group 2 pollen allergens, and has been hypothesized to function as a polysaccharide-binding domain, although this is yet to be proven experimentally [19,20]. Another group called expansin-like family X (EXLX) has been identified. This group comprises of proteins with distant homology to EXPAs and EXPBs, and are present in non-plant organisms [21]. Protein sequences with homology to expansins have been found in slime molds [22], bacteria [23,24], and ascomycete fungi [25,26]. Here, we report the identification and characterization of loosenin, a novel expansin-type protein, (LOOS1) from *Bjerkandera adusta*. Part of the loosenin sequence is similar to the DPBB domain present in plant expansins, and fungal β-1,4-endoglucanase family 45. The heterologously expressed LOOS1 preparations bind polysaccharides, permit sugar release from cellulose after treatment with a commercial cellulase and show loosening activity on cotton fibers. Finally, the recalcitrant natural lignocellulosic substrate *Agave tequilana *bagasse was 7.5 times more susceptible to the action of a cocktail of cellulases and xylanases after it had been previously treated with LOOS1.

## Results

### Cloning of *loos1 *gene

We were interested in finding novel cellulolytic and cellulose-disrupting activities of fungal origin. Upon analysis of 768 sequenced clones from a subtracted cDNA library from *B. adusta *(Cuervo *et al*; manuscript in preparation), one sequence, that we have termed *loos1*, was selected because it presented high identity to proteins from fungal species *Laccaria bicolor *[EMBL:B0CQ69] 64%, *Schizophyllum commune *[EMBL:D8QC43] 53%, and *Flammulina velutipes *[EMBL:ACZ59470.1] 54%, annotated as expansin family proteins (Additional File 1, Table S1 and Figure S1). We aimed to determine if *loos1 *was also expressed under lignocellulose growing conditions. cDNA was amplified by RT-PCR from total RNA, obtained from *B. adusta *grown on wheat straw medium. The PCR product was cloned and its sequence confirmed. The analysis of the 390 bp clone suggested that it encodes a novel type of protein with distant homology (approximately 20%) to the family of plant expansins, that we named loosenin [GenBank:GU322016].

**Figure 1:**
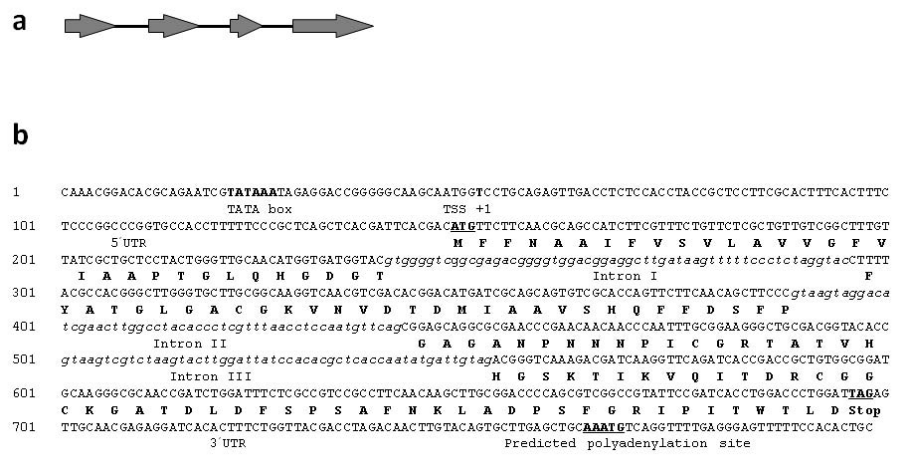
***loos1 *gene structure**. **(a)** Map of the *loos1 *gene organization. Exons are shown as arrows and introns as lines, drawn to scale. **(b)** The predicted core of the promoter region containing a TATA box at -25 is distinguished in large letters, as well as the nucleotide indicating the predicted site of transcription initiation (TSS). Untranslated regions, 5' and 3', and a possible polyadenylation signal are shown flanking the coding sequence of *loos1*. Introns I, II and III are shown in lowercase italic letters, to compare to the coding sequence of *loos1*.

*loos1 *genomic DNA sequence includes three introns I, II and III, two of which (introns II and III) exhibit the canonical 5'-GT....AG-3' donor-acceptor pairs. Intron lengths are 55, 53 and 52 nt, respectively, in agreement with the average intron size of filamentous fungi (50-70 bp), and account for 160 extra nucleotides relative to the cDNA (Figure 1a). The 5' and 3' UTRs are predicted to consist of 98 and 100 nucleotides respectively (Figure 1b).

### Homology Modeling of Loosenin

**Figure 2:**
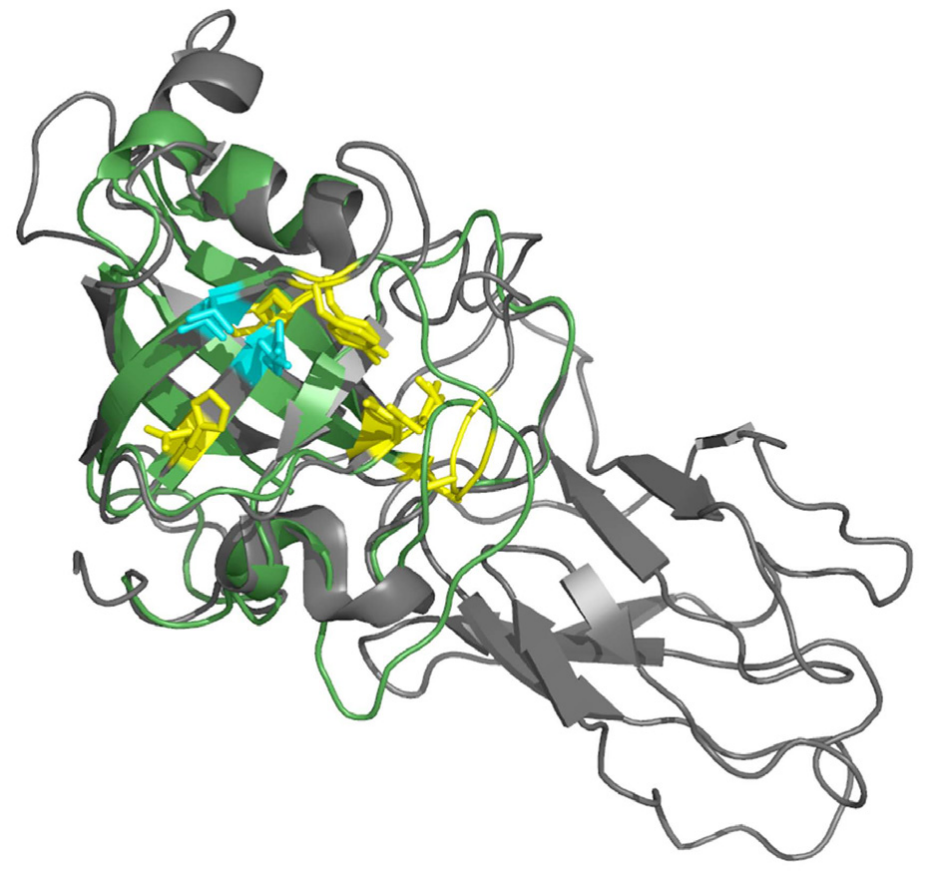
**Structural superposition of the Loosenin model (green) with **2hcz, **an EXPB (dark grey)**. The residues identified by Kerff et al as belonging to the polysaccharide binding groove in BsEXLX are indicated as sticks. Strictly conserved residues in Kerff's analysis are indicated in cyan and conserved residues in yellow. The structural alignment and figure were produced with Pymol.

The loosenin amino acid sequence was used for fold recognition using the PHYRE web server version 0.1. The top three results were EXLX1 from *Bacillus subtilis *[PDB:2BH0], the homologue pollen allergen PHL P1 N-terminal domain from *Phleum pratense *[PDB:1N10] and an EXPB and group-1 allergen from maize [PDB:2HCZ]. All had an estimated precision of 100% indicating a successful fold assignment to the DPBB fold family. Primary sequences identities were 19, 19 and 20% respectively confirming that loosenin is indeed a remote homologue of the expansins. We used the alignments provided by PHYRE to construct five models of the complete loosenin amino acid sequence with 2BH0, 1N10 and 2HCZ as templates using maximum MD-refinement. All five models were essentially identical with an average RMSD of 0.4 Å. Structures 2BH0 and 1N10 had an RMSD (of the equivalent superimposed alpha-carbons) of around 1.4 Å to the models when 2HCZ had an RMSD of 0.5 Å, although amino acid identity to loosenin was very marginally greater (Figure 2). This measure would reflect the similarity of the proteins cores. Unlike typical expansins, loosenin is composed of a single domain, albeit one highly similar to domain I of expansins, as evidenced by fold recognition. Kerff et al [27] performed a sequence analysis of EXLX1 (structure 2BH0) together with other polysaccharide recognizing proteins through which they identified several conserved residues. Similarly, we superimposed the loosenin model with the above-mentioned structures and identified the equivalent residues. In loosenin, T31 and D105 (highlighted in cyan in Additional File 1, Figure S2) correspond to the two strictly conserved residues, equivalent to T12 and D82 in EXLX1, and known to form a conserved hydrogen bond between the OH group of Thr and the carboxylic group of Asp. Other residues that show perfect sequence conservation between loosenin and the other three structures are G38, A39, G75, T92, and D93. Four more positions identified as important by Kerff et al correspond to loosenin Y33 (also conserved in 2HCZ and 1N10, however it is substituted by a T in 2BH0); A52 (conserved in 2BH0, but found as C in the other two structures); D103 (is substituted by A in 2BH0, or H in 1N10 and 2HCZ); and finally F109 (found conserved in the rest of the structures as an L). Except for G75, all these residues were identified by Kerff et al. as part of the groove which is thought to serve as the polysaccharide binding site by means of hydrogen bonding. The model thus suggests that loosenin is also a polysaccharide binding protein.

### Heterologous expression of Loosenin in *Saccharomyces cerevisiae*

**Figure 3:**
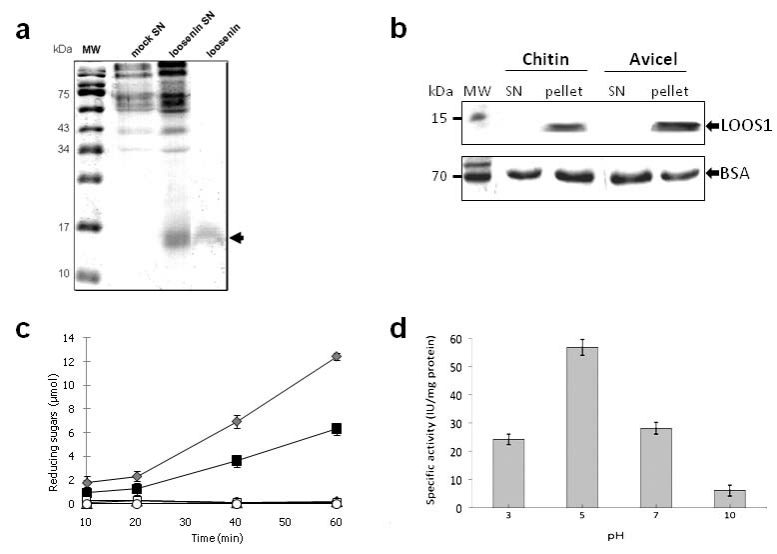
**Loosenin enrichment, activity and binding to polysaccharides**. **(a)** LOOS1 enrichment. SDS-PAGE of proteins from yeast culture supernatants transformed with empty vector pSAL3 (mock SN), pSAL3-*loos1 *(loosenin SN), and enriched LOOS1 (loosenin). **(b)** LOOS1 binds specifically to Avicel (crystalline cellulose) and chitin. The bounded (pellet) and unbounded (SN, supernatant) fractions are shown (top panel). BSA on the other hand, is found in both fractions, indicating unspecific interaction with the matrixes (lower panel). **(c) **RS release by cellulase from cotton fibers previously incubated with: two different concentrations of loosenin plus cellulase (20 μg black squares, 40 μg gray diamonds); acetate buffer (open triangles); acetate buffer plus cellulase (gray circles); mock SN plus cellulase (open squares); and only LOOS1 (open circles). **(d) **Specific activity of cotton fibers previously treated with LOOS1, at different pH, plus cellulase. All experiments were performed in triplicate, and error bars indicate the standard deviations.

To determine if loosenin affects cellulose, *loos1 *cDNA was cloned and heterologously expressed in *Saccharomyces cerevisiae *under a regulatable Cu-responsive promoter. The recombinant LOOS1 was secreted and enriched preparations were obtained by ultrafiltration from culture supernatants (Figure 3a). Preparations of crude extracts and enriched protein showed that loosenin molecular weight is approximately 15 kDa when expressed in yeast (Figure 3a), suggesting some type of post-translational modification, given the predicted 11.4 kDa molecular weight of the mature protein.

### LOOS1 is a polysaccharide binding protein

Since LOOS1 contains a potential polysaccharide binding groove, we investigated its binding ability to two different substrates: crystalline cellulose (Avicel) and chitin. Indeed, LOOS1 was only found in the precipitated fraction after incubation with the substrates (Figure 3b). Binding of BSA to the same substrates was non-specific and clear bands can be observed on both, the precipitated and the soluble fractions (Figure 3b).

### Loosenin allows endoglucanase activity on commercial cotton fibers

To explore the susceptibility of loosenin-treated cotton fibers to the hydrolytic action of enzymes that act on amorphous cellulose, a commercial preparation of cellulase corresponding principally to endoglucanase (E.C 3.2.1.4, as experimentally confirmed) (Additional File 1, Figure S3), was added to loosenin-treated cotton fibers. Reducing sugars (RS) release was achieved in the presence of cellulase at a velocity of 1.12 μmol per minute when the fibers had been previously treated with 20 μg of loosenin (Figure 3c), for a specific activity of 56 IU/mg/protein. The velocity of RS release was directly proportional to the amount of loosenin added to the reaction mixture (2.19 μmol/min when 40 μg of loosenin were added [Figure 3c]), and specific activity of 54.75 IU/mg protein). This effect is specific to the presence of LOOS1, given that addition, at the same concentrations, of proteins from mock supernatants of *S. cerevisiae *cultures or an irrelevant protein (BSA) produced no activity after treatment with cellulase (Figure 3c; and Additional File 1, Figure S4). Additionally, although mercerization allowed higher levels of RS release from loosenin treated fibers in comparison to non-mercerized fibers (compare Figure 3C with Additional File 1, Figure S5a) no RS release was detected in the untreated control, indicating that mercerization treatment does not disrupt the fibers to allow sugar liberation by cellulase (Figure 3c). Finally, lack of RS release in the absence of cellulase indicates that loosenin has no cellulolytic activity *per se*.

### Thermal- and pH-stability of loosenin

Since robustness of enzymes is a key factor for industrial applications, the analysis of the thermal- and pH-stability of LOOS1 was carried out. Thermostability of LOOS1 was analyzed by incubating the reaction of loosenin and cotton fibers at 15, 25, 40, 60 and 80°C, and then adding cellulase to monitor the release of RS at 50°C. Specific activity at 25°C was 55.58 IU/mg protein. No RS were detected after treatment at 40°C and higher temperatures, indicating than LOOS1 activity is not thermostable (data not shown). Similarly, the effect of incubating LOOS1 for 8 h in buffers with pH ranging from highly acidic to highly alkaline was analyzed. In this case, at pH 3, LOOS1 retained 42.6% of its original activity (24.17 IU/mg compared to 56.7 IU/mg achieved at pH 5). At neutral pH LOOS1 remaining activity decreased to 49.5%, and to 10.6% at pH 10 (Figure 3d).

### Loosenin allows digestion of a natural lignocellulosic material

**Figure 4:**
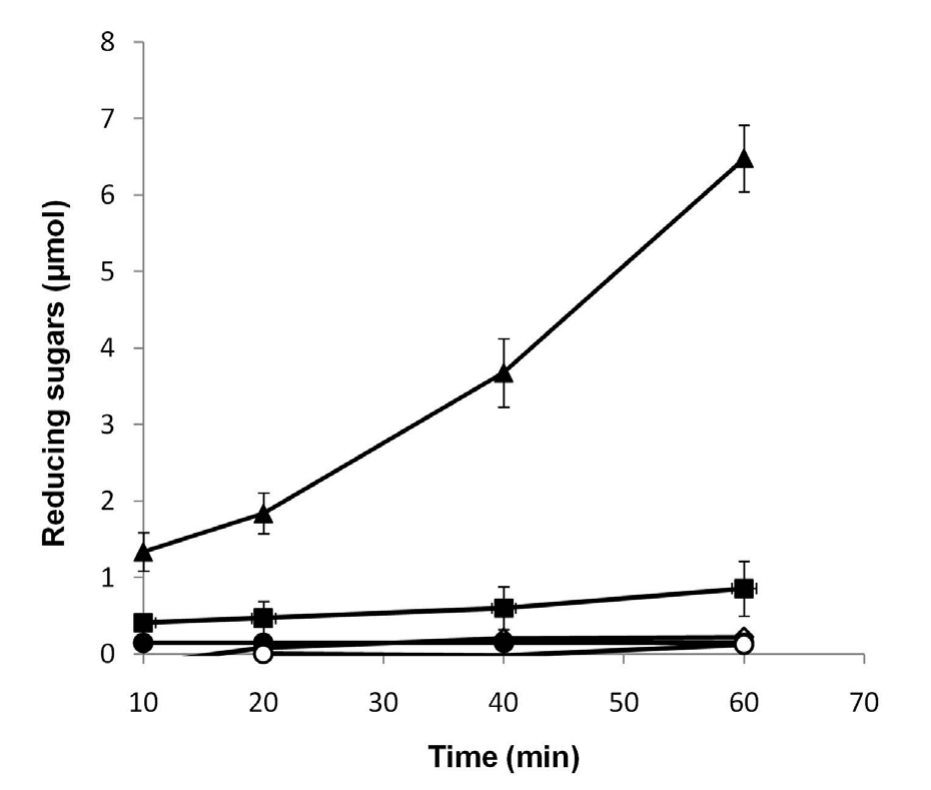
**Loosenin treatment of *Agave tequilana *bagasse**. Washed *A. tequilana *bagasse was cut into pieces and then incubated with 50 mM acetate buffer, 50 mM acetate buffer plus 20 μg of loosenin, or 20 μg of proteins from mock supernatant during 8 h at room temperature. Then, one μl of GC 220 (equivalent to 1 U of endoglucanase) was added and samples taken at the indicated time points to analyze for RS release. Acetate buffer (diamonds); acetate buffer + GC 220 (squares); mock SN [20 μg] + GC 220 (open circles); loosenin [20 μg] (closed circles); loosenin [20 μg] + GC 220 (triangles). All experiments were performed in triplicate, and error bars indicate the standard deviations.

We also analyzed the effect of incubating *Agave tequilana *fibers with loosenin and then treatment with a commercially available mixture of cellulases and xylanases (GC 220). After 10 min of incubation with GC 220, loosenin-treated agave fibers samples contained 3.2 times more RS than samples treated with GC 220 only, and by one hour of incubation loosenin-treated samples contained 7.5-fold more RS than the untreated samples (Figure 4).

### Loosenin exhibits disrupting-activity on cellulose

**Figure 5:**
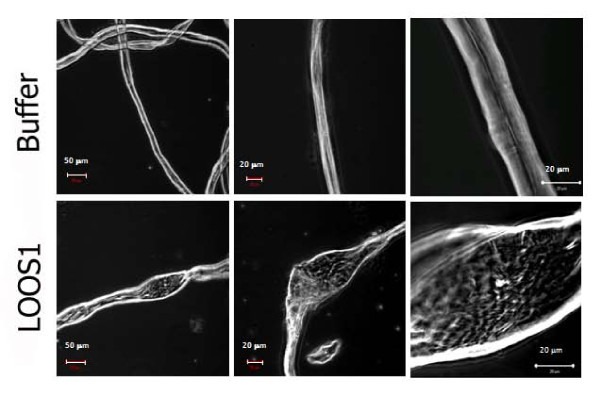
**Representative micrographs of cotton fibers incubated with acetate buffer pH 5 (top panels), or loosenin (lower panels), at three different optical magnifications (20× left, 40× middle and 100× right panels)**. Bubble-like structures are observed in the LOOS1-treated samples.

Loosenin disruptive-activity on cotton fibers was then investigated. Cotton fibers incubated for 8 h with 20 μg LOOS1, mock supernatant proteins or acetate buffer only were visualized by phase contrast microscopy. The cotton fibers in the untreated controls had a homogeneous structure with widths between 13 and 17 μm along the fiber (Figure 5). On the contrary, LOOS1-treated fibers showed local disruption of the fiber structure observed as enlarged "bubbles" (defined as those loosened structures measuring at least 40 μm width; Figure 5), 2.6 times wider with respect to the untreated controls. The occurrence of these bubbles also depended on the concentration of loosenin added, being observed at a frequency of 1 bubble/field at 20 μg (average width: 52.25 μm ± 10.07), and 1.6 bubbles/field at 40 μg (average width: 66.62 μm ± 10.62), compared to the absence of evident bubbles in the untreated control (which occasionally showed wider regions of up to 30 μm, average width: 26.60 μm ± 4.21) (Figure 5). Loosened areas of cellulose have been previously reported for fungal swollenins that show expansin-like activity [26].

## Discussion

Cellulose recalcitrance remains a limiting factor for enzymatic hydrolysis in biofuels production. Increasing of the loosened cellulose surface area by the use of non-hydrolytic proteins (a process called amorphogenesis) would allow access to hydrolytic enzymes making the saccharification process more efficient. We have cloned and characterized a new type of expansin-related protein, loosenin, from the basidiomycete fungus *Bjerkandera adusta *that seems to have a role in amorphogenesis. This is the first report of a protein with expansin-like activity for a basidiomycete. Indeed, Blast similarity searches recovered fungal sequences annotated as members of the expansin superfamily. Despite the low primary sequence similarity of LOOS1 to plant expansins (approximately 20%), its three-dimensional model fits domain I of the crystallized EXLX1 from *Bacillus subtillis*, *Zea mays *β-expansin and PHL P1 homologue with the highest score possible, and conserves those residues thought to be involved in binding to the polysaccharide chain.

Interestingly, the structure of loosenin consists of only one domain with a DPBB fold, the first reported of this type, suggesting that this structure might be sufficient to produce cellulose-disrupting activity. Expansin domain II appears to bind carbohydrates via a number of hydrophobic residues that tightly associate with the substrate, given the need of local (cell specific) cell wall disruption. For loosenin, both binding and disrupting activity reside in domain I. On the other hand, expansin domain II could serve other purposes, such as providing higher activity or stability. It was also noticed by Kerff et al, that the potential polysaccharide binding groove of domain I is extended by three aromatic residues on the surface of D2, allowing the binding of four additional saccharide units, suggesting a stronger union to the polysaccharide and possibly increasing its activity towards it. Loosenin is a good model to answer these questions by making fusions to homologues of expansin domain II.

Our work demonstrates that, in accordance with the prediction of the three-dimensional modeling, LOOS1 binds specifically to crystalline cellulose and chitin. Furthermore, loosenin is indeed a cellulose-disrupting protein, and that cotton and Avicel (which behaved in the same way as cotton when mercerized or not in the presence of loosenin [Additional File 1, Figure S5b and S5c]), are modified by its action. Indeed, dose-dependent LOOS1 treatment of cotton fibers induces local disruptions that possibly become a substrate for an endoglucanase enzyme that otherwise could not act upon them, supporting its role as a protein involved in amorphogenesis. It is important to remark that loosenin, contrary to plant expansins, was successfully heterologously expressed in yeast, given the industrial significance of cellulose-disrupting proteins and cellulolytic enzymes. The putative secretion signal sequence of loosenin seems to be recognized by *S. cerevisiae *because the protein was found in the culture supernatants. The activity of loosenin purified from *B. adusta *itself remains to be analyzed and compared to that reported here, given that it is known that *S. cerevisiae *hyperglycosylates secreted proteins in some cases (N-glycosylation). Indeed, loosenin molecular weight was higher than the predicted by its primary sequence (15 versus 11.4 kDa) suggesting a posttranslational modification. In this respect, three O-glycosylation sites were predicted at positions 3, 105 and 107, but no N-glycosylation sites were found for the loosenin of *B. adusta*. Further experiments are needed to understand the role of glycosylation on loosenin activity.

The biological function of loosenin in *B. adusta *remains to be analyzed. It is possible that the fungus uses this protein to efficiently degrade lignocellulose together with a battery of extracellular hydrolytic enzymes (ligninases, cellulases, xylanases, etc). However, a role of loosenin in the physiology of the fungus cannot be ruled out, as it could also participate in the remodeling of the fungal cell wall to allow hyphal growth as suggested for *Aspergillus nidulans *EglD with an expansin-like motif [25].

Finally, loosenin represents a good candidate as an additive to enhance sugar production from plant biomass. Loosenin activity was more efficient when the lignocellulosic materials were mercerized, as seen for other saccharification processes. *Agave tequilana *is a crop extensively grown in some areas of Mexico, and the shredded fibrous waste is usually burnt or left to decompose. Indeed, *A. tequilana *fiber became a susceptible substrate for a cocktail of commercial cellulases and xylanases in the presence of LOOS1. Loosenin shows optimum activity at the same pH as most cellulolytic enzymes. And although it is not a thermostable protein, probably because of the temperate origin of *B. adusta*, similar sequences are present in a number of other basidiomycetes fungi, opening the possibility to find them expressed also in thermophilic species, such as *Pycnopourus sanguineus *[28], or others. It is important to remark upon the low enzymatic activity needed in combination with loosenin to observe its effect, given the high costs of enzymes at industrial levels.

## Conclusions

Here we describe a new type of protein with a role in amorphogenesis of cellulose that we have called loosenin, from the basidiomycete *Bjerkandera adusta*. Loosenin is distantly related to plant expansins, binds to cellulose and chitin, and has a disrupting activity on cellulose fibers. Treatment of lignocellulosic materials (cotton fibers and *Agave tequilana *bagasse) with loosenin enhances sugar liberation after the addition of commercial cellulases, suggesting an interesting potential use for the production of fermentable sugars from lignocellulose.

## Methods

### Strains and growth conditions

*Bjerkandera adusta *strain UAMH 8258 was kindly provided by Dr. Rafael Vazquez-Duhalt. Mycelium was grown on PDA medium (2% potato, 2% dextrose and 1.5% agar) for its propagation and stocking. For the isolation of *loos1 *gene, *B. adusta *was grown in 2% wheat straw liquid medium (mineral base medium: 7.8 mg/L CuSO_4_·5H_2_O, 18 mg/L FeSO_4_·7H_2_O, 500 mg/L MgSO_4_·7H_2_O, 10 mg/L ZnSO_4_, 50 mg/L KCl, 1 g/L K_2_HPO_4 _and 2 g/L NH_4_NO_3_, 1.5% agar; pH 5 (modified from [29]) supplemented with powdered wheat straw (maximum and minimum particle sizes of 3 and 0.5 mm), for 5 days at 28°C and 200 rpm.

*Saccharomyces cerevisiae *strain W303a (*MATα can1-100 ade2-1 his3-11, 15 leu2-3, 12 trp1-1 ura3-1*) was grown in SC-Ura-minus medium [0.67% yeast nitrogen base (Difco), 2% dextrose (Baker), adenine 20 mg/L, leucine 60 mg/L, tryptophan 20 mg/L and histidine 20 mg/L (Sigma-Aldrich)]. For heterologous expression of loosenin, SC-Ura-minus medium was supplemented with 50 μM Cu_2_SO_4 _and cells were grown at 28°C for 1 day.

*E. coli *strain DH5α was used for plasmid propagation and manipulation according to [30].

### Molecular cloning of loos1

*loos1 *sequence was originally identified by BLAST analysis (Additional File 1, Table S1) of clones obtained from a subtracted cDNA library from *B. adusta *grown in the presence of crude oil (Cuervo, *et al*, manuscript in preparation). The sequence was then amplified from a sample of total RNA of *B. adusta *grown in wheat straw medium by Reverse Transcription coupled-PCR with primers LOOS1fwd: 5'-CGGAATTCATGTTCTTCAACG-3' and LOOS1rev: 5'-CCTCGAGCTAATCCAGGGT-3'. The primers sequences included *Eco*RI and *Xho*I sites (underlined) at the 5'- and 3'-ends, respectively, to facilitate subsequent cloning steps. The 390 bp PCR fragment was purified and cloned into the pGEM-T (Promega) vector resulting in pGEM-LOOS1, and its sequence was confirmed (Macrogen, USA).

### Genomic DNA extraction

Mycelium grown for 7 days in liquid wheat straw medium was collected, frozen and pulverized with liquid nitrogen. Genomic DNA was extracted with UltraClean Megaprep Soil DNA kit (MoBio), and used as template for PCR amplification with primers LOOS1fwd and LOOS1rev. The PCR product was cloned in plasmid pGEM-T (Promega) resulting in vector pGEM-LOOS1 g, and its sequence was determined (Macrogen USA).

### Domain prediction

Conserved domains were identified using the CDD (Conserved Domain Database, http://www.ncbi.nlm.nih.gov/Structure/cdd/wrpsb.cgi) and InterProScan (http://www.ebi.ac.uk/Tools/InterProScan/) databases.

### Structural Modeling of Loosenin

We identified templates for Loosenin homology modeling using the PHYRE Protein Fold Recognition Server [31]. This program identified two expansins and one expansin-homologue with the highest scores (100% estimated precision). We then modeled the Loosenin sequence using the program modeller9v7 [32] using the sequence alignments obtained by PHYRE simultaneously generating 5 models with a molecular dynamics level of refine.very_slow. The four resulting models were compared using Pymol (DeLano Scientific LLC), first by fitting them together and then comparing the structures.

### Expression of recombinant LOOS1 in yeast

Plasmid pGEM-*loos1 *was digested with *Eco*RI and *Xho*I and the 390 bp resulting fragment was subcloned in vector pSAL3 [33], giving rise to plasmid pSAL3-*loos1*. Yeast strain W303α was transformed with plasmid pSAL3-*loos1 *or empty pSAL3 by the lithium acetate method [34], and selected on solid SC-Ura-minus medium. After 3 days of incubation, one colony of each transformation was grown in a pre-inocule of 20 ml of SC-Ura-minus medium at 28°C and 250 rpm to mid log-phase (between 0.4-0.6 OD), at this point a dilution was made to 0.1 OD in 1 L of SC-Ura-minus medium, adding Cu_2_SO_4 _to a final concentration of 50 μM, and supplementing the media with protease inhibitors according to the manufacturer's instructions (Complete^® ^Roche). Finally, cells were grown to 0.8 OD to recover supernatant by centrifugation (1073 × *g *for 10 min at 4°C), that was kept at 4°C until use.

### Loosenin enrichment

Supernatant recovered from 1 L yeast cultures was concentrated 50 times by ultrafiltration through a 10 kDa cut-off membrane (Amicon stirred cell 8400, and Ultracell membranes, Millipore), and then filtered through a 30 kDa cut-off membrane (Amicon Ultra 4 Centrifugal Filter, Millipore). The filtrate was recovered and concentrated again through 10 kDa cut-off membranes (Amicon Ultra 4 Centrifugal Filter, Millipore) to a final volume of 200 μl. Mock supernatant from empty pSAL3 vector cultures was only concentrated by ultrafiltration (10 kDA cut-off). Protein concentration was measured according to [35] using a bovine serum albumin (BSA) standard curve. Enrichment and molecular weight estimations of recombinant loosenin were performed resolving 20 μg of protein in 15% SDS-polyacrylamide gels. 20 μg of mock supernatant were loaded as well. The PA gel was stained with 0.25% Coomassie Blue R-250 (Sigma-Aldrich) and distained with a solution of methanol:water and acetic acid. Treatments of samples with loosenin were performed with these enriched preparations throughout this work.

### Polysaccharides binding assay

The assay was performed as reported by [36] with some modifications. Avicel PH-101 (Fluka) and chitin from shrimp shells (Sigma-Aldrich) were used as binding matrices. 40 μg of LOOS1 or BSA were mixed with 50 mg of binding matrix suspended in 200 μl of 15 mM phosphate buffer (pH 7.4) containing 150 mM NaCl. The mixture was incubated for 30 min with agitation, and then the supernatant was removed by centrifuging at 13,500 rpm/2 min/RT (unbound fraction). Pellets were washed 4 times with the same buffer; the protein was dissociated from the matrix by boiling with SDS-PAGE sample buffer (bound fraction). Both fractions were loaded in a 15% polyacrylamide gel and resolved by electrophoresis. Gels were stained with Coomassie blue.

### Loosenin treatment of cotton fibers

Pharmaceutical-grade cotton fibers were mercerized according to [26]; briefly, 7 mg of cotton fibers were incubated with 25% NaOH for 15 min at 4°C, and washed several times with distilled water to eliminate the excess of alkali. Loosenin and control treatments of the fibers were as follows: incubation with (a) 20 or 40 μg of loosenin in 1 ml of 50 mM acetate buffer pH 5 for 8 h at 25°C; (b) 20 or 40 μg of proteins from mock supernatants, (c) buffer only, (d) 0.5 U of commercial *Trichoderma viride *cellulase (Sigma-Aldrich Cellulase E.C 3.2.1.4, Catalogue no. C-1794). For microscopic observations these reaction mixtures (a-c) were sonicated (Ultrasonic cleaner 1510, Branson) for 1 min, after incubation and the cotton fibers visualized by contrast phase microscopy (Zeiss).

### Reducing sugars release assay

Commercial *T. viride *cellulase was added to final concentration of 0.5 U to the above reaction mixtures (a-c) after 8 h of incubation at 25°C, and the temperature increased to 50°C. Aliquots of 50 μl were then taken at 10, 20, 40 and 60 min after the addition of cellulase. Concentration of released reducing sugars was determined using the DNS method described previously [4]. All assays were performed in triplicate. Specific activity of loosenin was indirectly calculated by dividing the amount of RS release after the loosenin/cellulase treatment by the amount of loosenin protein used in the assay that although non-hydrolytic, should indirectly reflect the degree of disorganization of the fiber.

### Thermal- and pH-stability of loosenin

Heat-stability of loosenin was assayed by incubation of mercerized cotton as explained before except that loosenin-treatment reactions (or control reactions) were carried out at 40, 60 or 80°C; treatment was ended by placing the samples on ice, and then incubated at 50°C after the addition of cellulase. Quantification of RS release was then performed as explained above. Similarly, pH-stability determination was performed in reactions at pH ranging from 3 to 10, followed as well by quantification of RS release at pH 5. System buffers were McIlvaine (pH 3), sodium citrate (pH 5 and 7), and borate-HCl (pH 10).

### Treatment of a natural lignocellulosic substrate

*Agave tequilana *fiber was cut with scissors into 2.5-5 mm pieces, and washed with distilled water overnight and five consecutive times, after which no RS release were detected by the DNS method. Then, 25 mg of these fibers were mercerized as explained above, and then incubated 8 h at 25°C with 20 μg of LOOS1 or with 20 μg of proteins from mock supernatant in 1 ml 50 mM acetate buffer pH 5. Next, 1 μl of commercially available enzyme cocktail GC 220 (Genencor-Danisco) containing cellulases and xylanases activities, was added to the reaction mixture (equivalent to 40 μl/g fiber and to 1 U of endoglucanase, as determined experimentally), and incubated at 50°C, to analyze for RS release through time as explained before.

## Competing interests

The authors declare that they have no competing interests.

## Authors' contributions

REQ-C performed the experiments and drafted the manuscript. CM-A assisted with data analysis and manuscript preparation. LC-S analyzed and identified the loosenin sequence in a set of sequences obtained from a cDNA library. LS participated in the bioinformatics analysis. JF-M conceived of the study, and participated in its design and coordination and helped to draft the manuscript. All authors read and approved the final manuscript.

## Supplementary Material

Additional file 1 **Supplementary Information**. Supporting data for this work. Click here for file
