# Supplementary material for: Loosenin, a novel protein with cellulose-disrupting activity from Bjerkandera adusta
Source: Microb Cell Fact. 2011 Feb 11;10:8. doi: 10.1186/1475-2859-10-8 (PMC3050684; doi:10.1186/1475-2859-10-8)
Supplement: Additional file 1 — Supplementary Information. Supporting data for this work. [file 1475-2859-10-8-S1.RTF]

Supplementary Information
Contents: supplementary Table 1, and supplementary figures 1 through 5
Loosenin, a novel protein with cellulose-disrupting activity from Bjerkandera adusta.
Rosa E Quiroz-Castañeda, Claudia Martínez-Anaya, Laura I Cuervo-Soto, Lorenzo Segovia and Jorge L Folch-Mallol

Supplementary Table 1. Most similar amino acid sequences to LOOS1.
Accession Number	Annotated activity [Organism]	Identity	

[GenBank:GU322016]	
loosenin [Bjerkandera adusta]		
[NCBI:XP_001873229.1]	expansin family protein [Laccaria bicolor S238N-H82]	64%	
[GenBank:BAH80450.1]	putative riboflavin aldehyde-forming enzyme [Lentinula edodes]	57%	
[NCBI:XP_002912124.1]	riboflavin aldehyde-forming enzyme [Coprinopsis cinerea okayama7#130] 	57%	
[NCBI:XP_003029770.1]	Non-Catalytic module family EXPN protein [Schizophyllum commune H4-8]	53%	
[GenBank:EEQ45357.1]	conserved hypothetical protein [Candida albicans WO-1]	52%	
[NCBI:XP_002419772.1]	unnamed protein product [Candida dubliniensis CD36] 	52%	
[NCBI:XP_002545265.1]	predicted protein [Candida tropicalis MYA-3404]	50%	
[NCBI:XP_001524386.1]	conserved hypothetical protein [Lodderomyces elongisporus NRRL YB-4239] 	50%	
[NCBI:XP_460115.2]	DEHA2E18744p [Debaryomyces hansenii CBS767] 	52%	
[NCBI:XP_001387493.1]	riboflavin aldehyde-forming enzyme [Scheffersomyces stipitis CBS 6054] 	56%	
[NCBI:XP_001259643.1]	riboflavin aldehyde-forming enzyme [Neosartorya fischeri NRRL 181]	42%	
[GenBank:ACZ59470.1]	expansin family protein [Flammulina velutipes]	54%	
[NCBI:XP_001274340.1]	riboflavin aldehyde-forming enzyme [Aspergillus clavatus NRRL 1] 	48%	
[NCBI:XP_002842519.1]	hypothetical protein [Tuber melanosporum Mel28]	51%	
[NCBI:XP_753596.1]	riboflavin aldehyde-forming enzyme [Af293] 	49%	


Figure S1. LOOS1 protein sequence was used for a pBLAST search of similar sequences. Of the twenty most similar hits, five were discarded because of redundancy, the rest of the sequences were aligned with Clustal 2.0.12. Strictly conserved residues are marked with an asterisk, symbols . or : indicate equivalent positions. Boxes show the 11 residues thought of forming the polysaccharide binding shallow groove of expansin B. subtilis EXLX1 (color coding is same than Figure 3).


Figure S2. Sequence alignment of the three-dimensional structure of templates [PDB:1N10, 2BH0 and 2HCZ] with Loosenin model. Polysaccharide binding conserved residues are colored with same code than Figure 3. β-strands and α-helices are marked with blue and red boxes, respectively. 


Figure S3. Analysis of the hydrolytic activity of the C1794 Commercial cellulase preparation. 20 ug of Avicel (microcrystalline cellulose, open symbols) and CMC (amorphous cellulose, closed symbols) were incubated with increasing concentrations of cellulase C1794 (0.5, 5 and 50 U) and release of RS analyzed. CMC incubated with 0.5 U of enzyme shows RS release. Higher enzyme concentrations produce an amount of RS already close to saturation at the conditions tested. Avicel is not a substrate at any of the enzyme concentrations used, indicating a negligible cellobiohydrolase activity in this preparation. All experiments were performed in triplicate, and error bars indicate the standard deviations.


                  
Figure S4. Modification of cotton fibers is specific to treatment with Loosenin. Cotton fibers were incubated with 20 μg of a control protein (BSA) and increasing concentrations (0, 0.5, 5 and 50 U) of endoglucanase to analyze RS release. All experiments were performed in triplicate, and error bars indicate the standard deviations.

                            
Figure S5. Mercerization increases LOOS1 activity. Non-mercerized cotton fibers (a), mercerized Avicel (b), and non-mercerized Avicel (c), were incubated with: acetate buffer (cross), acetate buffer and endoglucanase (triangles), loosenin [20 μg] (squares), and loosenin [20 μg] plus 0.5 U endoglucanase (diamonds) to analyze for release of RS. All experiments were performed in triplicate, and error bars indicate the standard deviations.
